# Causes of intraprocedural discomfort in colonoscopy: a review and practical tips

**DOI:** 10.1177/26317745241282576

**Published:** 2024-10-05

**Authors:** Jabed F. Ahmed, Ara Darzi, Lakshmana Ayaru, Nisha Patel

**Affiliations:** Endoscopy Department, Imperial College Healthcare NHS Trust, St Marys Hospital, Praed St, London, W2 1NY, UK; Department of Surgery & Cancer, Imperial College London, London, UK; Department of Surgery & Cancer, Imperial College London, London, UK; Gastroenterology Department, Imperial College Healthcare NHS Trust, London, UK; Gastroenterology Department, Imperial College Healthcare NHS Trust, London, UK; Department of Surgery & Cancer, Imperial College London, London, UK

**Keywords:** colonoscopy, discomfort, endoscopy factors, novel solutions, pain, patient factors

## Abstract

Colonoscopy is a commonly performed procedure in the United Kingdom and the gold standard for diagnosis and therapy in the gastrointestinal tract. Increased levels of pain during colonoscopy have been associated with reduced completion rates and difficulties in maintaining attendance for repeat procedures. Multiple factors play a role in causing discomfort intra-procedurally: patient factors, such as gender, anatomy and pre-procedure anxiety; operator factors, such as patient position and level of experience and other factors, such as bowel preparation and total procedure time. A literature search was performed to identify papers that explained how patient, operator and endoscopy factors influenced pain and discomfort in endoscopy. A further search then also identified papers describing solutions to pain and discomfort that have been explored. After review of the literature, key methods are selected and discussed in this paper. Solutions and aids that can resolve and improve pain and discomfort include endoscopic methods such as variable stiffness and ultrathin scopes. Operator improvements in techniques and ergonomics alongside the use of newer technologies such as propelled endoscopy, computer-assisted endoscopy and task distraction. To improve patient experience and outcomes, the investigation and research into improving techniques to reduce pain is crucial. This review aims to identify the modifiable and non-modifiable factors associated with intra-procedural discomfort during colonoscopy. We discuss established methods of improving pain during colonoscopy, in addition to newer technologies to mitigate associated discomfort.

## Introduction

Colonoscopy is a commonly performed procedure in the United Kingdom and the gold standard for diagnosis and therapy in the gastrointestinal (GI) tract. Indications include bowel cancer screening, inflammatory bowel disease assessment, investigations for GI symptoms and polyp surveillance.^1^ Colonoscopy carries a low risk of morbidity with less than 0.1% perforation and a major bleeding rate.^2^ Despite this, pre-procedure screening with patients has placed the highest importance on the comfort experienced.^3^ In both sedated and unsedated patients, pain and discomfort have been reported in 79.2% and 88.7% of cases, respectively.^4^ In one study of 190 patients, severe pain was reported in as many as 20% of unsedated cases, and conversely, no discomfort was experienced to be as low as 5%.^5^

Increased levels of pain during colonoscopy have been associated with reduced completion rates and an unwillingness to undergo repeat or surveillance procedures resulting in increased ‘did not attend’ rates.^6^ Pain during colonoscopy could be linked with reduced diagnostic sensitivity due to reduced engagement with screening and surveillance services.^7^

To improve patient experience and outcomes, the investigation and research into improving techniques to reduce pain is crucial. Discomfort in colonoscopy can be pre-procedural such as related to bowel preparation, intra-procedural or post-procedural. This review focuses on intraprocedural pain and aims to identify the modifiable and non-modifiable factors associated with discomfort during colonoscopy. We discuss established methods of improving pain during colonoscopy, in addition to newer technologies to mitigate associated discomfort.

A literature search was undertaken on PubMed and Google Scholar looking at studies discussing pain and discomfort in colonoscopy. Key studies were then selected and discussed further in this review below. The methodology of the literature search is provided in the Supplemental Material.

## Mechanism of pain and discomfort during colonoscopy

**Figure 1.**
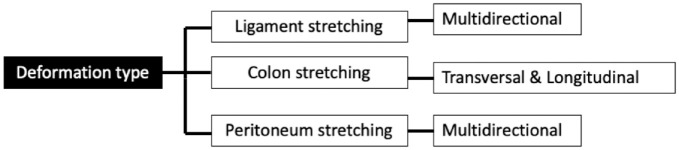
Deformation of the colon; types that occur in colonoscopy. Source: Adapted from Takahashi et al.^8^

When advancing a colonoscope, the colon wall, ligaments and peritoneum are invariably stretched and displaced from their natural structures.^8^ (Figure 1)

Sections of the bowel requiring increased ‘push forces’ to navigate include the sigmoid colon and hepatic flexures due to the increased angulation. This creates tension in the colon wall transversely and longitudinally, which resists further wall deformation. As a result, this resistance may force the colonoscope to bend or ‘loop’, which can commonly cause discomfort or pain.^9,10^

Intraluminal distension can elicit high-amplitude propagation peristaltic contractions in the bowel lumen. The exact stimulus for these contractions is not known, but it is theorised that distention resulting from insufflation with air or carbon dioxide is the trigger and that this majority of propagated contractions by distention is associated with pain.^11^ In addition, peritoneal folds are pain-sensitive to traction and stretching. Ligament stretching is hypothesised to cause pain as they are suspended from the flexures and consist of peritoneal folds.^8^

### Patient factors

There are a number of factors that contribute to discomfort or pain experienced during colonoscopy (Table 1). It is important to note the geographical effect on patients. The cultural perceptions can be different when it comes to expression of pain from various countries, particularly from west to east. Additionally, ethnicity may influence certain physical attributes that affect patient responses to the procedure.

**Table 1.** Summary of factors contributing to discomfort and pain experienced during colonoscopy.

The patient	Operator skill	Other factors
Age	Technique	Gastroscopy prior to colonoscopy
Gender		
Anatomy (Looping of the endoscope)	Torque steering	Total procedure time
BMI		
Indication	Level of experience	Bowel preparation
Past medical history	Patient position	
Pre-procedure anxiety		

Many studies have shown that younger age is associated with an increased risk of discomfort or pain during colonoscopy.^12^ This may be due to the diminishing perception of visceral pain in ageing patients. Older age, although not associated with increased pain, is associated with an increased likelihood of adverse events such as bowel perforation and bleeding.^13^ This may be due to increased intervention due to the increased incidence of large colonic polyps in the ageing population, which require removal.^14^

Gender-related differences in the perception of pain experienced during colonoscopy have also been observed. Many studies have shown that female gender is associated with increased complexity and discomfort.^15,16^ Anatomically, female colons are generally longer resulting in a longer procedural time.^17^ It has also been suggested loop formation risk increases particularly in the sigmoid due to the reduced muscle bulk and due to deeper, more rounded pelvises compared to males.^18^ Increased intrapelvic space in females, made greater with hysterectomy, increases the mobility of the sigmoid colon, this in turn increases the likelihood of looping and fixation of the colon.^19^

A recent study highlighted the response to pain may depend on social and cultural differences. These cohort of patients is at risk of underestimation of pain and is an important factor to highlight to endoscopists. Such groups in this cohort include male gender and normal body mass index (BMI).^20^

A low BMI is associated with discomfort, prolonged insertion time and incomplete procedures, independent of gender. It is proposed that lower BMI is also associated with lower muscle bulk and hence higher levels of loop formation within the bowel.^21^ A history of constipation has also been reported as a predictor of challenging colonoscopy. Constipation due to redundant colon may result in poor bowel preparation and hence a more challenging procedure. In contrast, diarrhoea has shown no link to discomfort and abdominal pain in colonoscopy.^15^ Anatomical variations of the bowel and mesentery may also influence pain experienced during a colonoscopy. The length of the colon and laxity of the mesentery, particularly for the ascending and descending colon, can result in increased bowel mobility and angulation. Tall patients and obese patients are reported to have longer splenic and hepatic bowel segments resulting in more difficult endoscopic negotiation.^22^

Previous abdominal and pelvic surgery may result in adhesion formation. Adhesional fixation of the colon increases the difficulty of colonoscopy insertion due to repeated and augmented mesenteric stretching of attached structures amplifying the levels of pain.^23^

Surgeries that result in the colon being shorter such as colectomy have reported no increase in discomfort; however, an increase in colonic or endoscopic angulation as a result can increase pain.^24^ Interestingly, a prospective study from Korea with an expert endoscopist using only intramuscular analgesia described a history of colorectal resection was inversely related to discomfort.^25^

Psychologically, undergoing a colonoscopy for some patients is a significant and potentially traumatic event. Prospective and retrospective studies have researched emotional processing showing if one can address catastrophising thoughts prior to the procedure, pain during endoscopy can be mitigated and the overall experience improved.^26^

Similarly, high levels of anxiety pre-procedure are also associated with discomfort as shown by a prospective survey of 118 patients.^27^

### Endoscopic factors

Modifications to the basic design of the endoscope have occurred over the years. Earlier models of the colonoscope are associated with significant patient discomfort, lower completion rates and increased perforation rate.^8^ There are modifications to endoscopes and aids that can be used in colonoscopy to minimise discomfort (Table 2). A wider field of view on endoscopes does not reduce pain, but a reduction in colonoscope diameter from 13.6 to 9.5 mm has improved flexibility and added to improving discomfort as shown in a study by one experienced endoscopist on 100 patients without sedation.^28^

**Table 2.** Summary of aids/solutions and modifications used in colonoscopy.

The endoscope	Operator skill	Aids used in colonoscopy
Loop resolution	Technique	Magnetic endoscopic imaging (MEI)
		Water immersion/exchange
Variable stiffness	Torque steering	Carbon dioxide insufflation
Thin/Ultrathin scopes	Level of experience	Abdominal pressure
		Sedation

Looping or bending of the colonoscope can occur due to a fixed segment of the colon, anatomical variants, long, redundant colons, adhesions or a particularly mobile colon. Looping occurs most commonly in the sigmoid colon but can occur anywhere along the length of the colon. It occurs in 91% of cases with the ‘N-sigmoid loop’ being the most common, occurring in 79% of cases.^25^ If the loop is not reduced, the colonoscope stretches and distends the colon and mesentery in response to the endoscopist forward pushing or insufflating resulting in additional pain. Loop reduction can help minimise pain experienced during colonoscopy. This has been significantly improved by the advent of navigational procedural tools such as Scope Guide (Olympus) and Scope Pilot (Pentax Medical), which allow recognition and resolution of loops in real-time.^29^

Variable stiffness is the mechanism of adjusting shaft stiffness using a central cable with a surrounding metal helical coil. Tension applied to the coil can stiffen and loosen the colonoscope. This allows improved avoidance of creating loops and discomfort during colonoscope insertion. Yoshikawa et al. performed a prospective study with 467 unsedated patients undergoing colonoscopy by experienced and less experienced endoscopists. The study concluded a significantly lower mean pain score with variable stiffness and also an improvement in pain score for the cohort of less-experienced endoscopists.^30^

Ultra-thin and small-calibre colonoscopes have been reported to be more helpful in reducing discomfort for older patients, patients with smaller colon diameters and females. They have greater flexibility compared to paediatric counterparts. A randomised control trial (RCT) with unsedated patients who could then request sedation/analgesia demonstrated in five patients where the endoscopist failed to complete the exam due to pain with the standard colonoscope, the ultrathin colonoscope was utilised. The second attempt was performed immediately, and caecal intubation was successful in all five cases.^31^

Other measures that are used for difficult colons that in turn reduce discomfort include overtubes and shape locking guides; these use a shape-locking mechanism and can switch between flexible and rigid structures in the sigmoid colon. Guide wire-assisted colonoscopy, single and double balloon enteroscopes and balloon catheters have also been recommended to improve discomfort in difficult colons from the American Society for Gastrointestinal Endoscopy technological committee.^32^

### Operator factors

Mastering the skill of colonoscopy is a fundamental pillar for any trainee in endoscopy and an integral way to minimise discomfort with good technique and improve patient’s experience and outcomes. This is reflected in the competencies required to achieve certification outlined and recently updated by the Joint Advisory Group (JAG) for trainees.^33^

**Figure 2.**
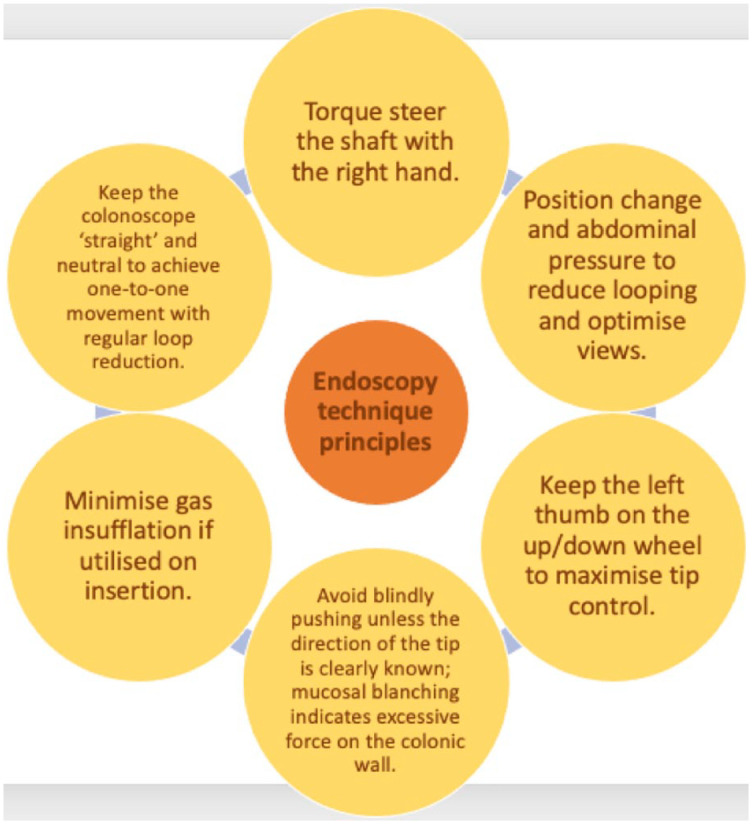
Endoscopy technique principles.^34^

Six principals recommended by Waye and Thomas-Gibson have remained relevant alongside the advancements in scope technology (Figure 2).^34^ Generally, endoscopists with a greater level of experience will perform a more comfortable procedure for the patient.^15^

Scope direction is influenced by the rotation of the shaft or torquing of the shaft, otherwise known as torque steering. Tip-up plus clockwise torque or tip-down plus anti-clockwise torque steers towards the right; and tip-down with clockwise torque or tip-up with anti-clockwise torque steers left. Combining these torque movements can help progress the scope in the colon and avoid the formation of loops.^34^

Patients’ position can also play a role in decreasing discomfort and also improving caecal intubation rate. Patients are usually commenced in the left lateral position, which allows for a digital rectal examination and early identification of rectal pathology. It has been suggested performing retroflexion at commencement when sedation may be at its deepest can be beneficial for patient comfort.^35^ Devitt et al. showed that a left lateral position was optimal for facilitating the passage of flatus and relieving bloating.^36^

Several studies have investigated the optimal starting position for colonoscopy. Zhao et al. conducted a two-centre randomised control trial involving 347 patients who underwent an unsedated colonoscopy either in the supine or the left horizontal position. The study concluded that supine starting position not only had a reduced pain score but also reduced the caecal intubation time.^37^ A systematic review of 2083 patient positions during colonoscopy was analysed with studies being performed with and without sedation. These included right versus left position, supine versus left position, prone versus left position and tilt-down versus left position. Each study on the right and supine positions reported reduced pain. However, prone and tilt-down positions did not significantly reduce pain and discomfort compared with the left position.^38^

Lin et al.^39^ also performed a meta-analysis of 644 patients and concluded no significant difference in pain from starting in different patient positions. Tilt-down starting position was analysed with studies reporting reduced pain, but this was not statistically significant.^40^

### Aids used in colonoscopy to reduce pain

Current sedation and analgesia used routinely in the United Kingdom are opiates (fentanyl) and benzodiazepines (midazolam) with some cases using general anaesthetic (propofol).^41^ Midazolam is often combined with fentanyl, the latter associated with significantly faster recovery times at lower doses (midazolam < 4 mg).^42^

The practice of administering and choice of sedation varies in different countries and is an important factor to note when assessing discomfort. In much of the world, the majority of colonoscopy is done under propofol sedation and would be considered painless as the patient is unconscious. In the United Kingdom, the emphasis is on conscious sedation. This holds certain advantages including improved mucosal visualisation as the patient can still follow instructions to change position, patients unsuitable for general anaesthetic can still undergo colonoscopy, reduced recovery time and hence faster turnaround time for the patient in the endoscopy department.^43^

Propofol is considered superior to midazolam and fentanyl. A meta-analysis of RCTs by Dossa et al. compared propofol versus midazolam and reported higher patient satisfaction with propofol. The average midazolam doses of included trials ranged from 2.9 to 7.6 mg and fentanyl from 50 to 154 mcg.^44^ A recent RCT by Kim et al.^45^ also found propofol to be superior to bolus or titrated midazolam given at a dose of 4.4–4.8 mg. An RCT of 100 patients comparing propofol versus midazolam and fentanyl reported propofol patients reached full recovery post procedure and were discharged sooner, but no significant improvement in pain was reported compared to midazolam and fentanyl given jointly.^46^

Patient-controlled analgesia (PCA) is a format of sedation delivery also being explored. A randomised double-blind study found PCA to have high satisfaction and willingness for a repeat with the same administration.^47^ An RCT from 2010 showed non-inferiority with PCA to standard sedation with a non-significant result showing lower pain scores.^48^

Antispasmodics such as hyoscine-*N*-butyl-bromide are commonly used to improve visualisation of the bowel mucosal surface on withdrawal and have been shown to improve patient discomfort. Sanagapalli et al. reviewed various antispasmodics in colonoscopy.^49^ In eight randomised control trials using hyoscine in colonoscopy only one reported a significant improvement in discomfort. The study suggested an increased benefit in patients who suffer from colonic spasms, thus resulting in improvement discomfort. It was also theorised that the greater central nervous system effect of hyoscine may result in greater doses of sedation and also increased chance of memory loss. It should be noted the randomised control trials analysed used air rather than CO2 insufflation. In addition, sedation was variable across the studies which would be a significant confounder including one study which used patient-controlled analgesia.

Endoscopy involves distention of the lumen using air or carbon dioxide insufflation to obtain adequate views during colonoscopy. This can however result in pain due to stretching of the mesentery.^50^ A randomised control trial using propofol as sedation comparing air to CO_2_ insufflation reported significant reduction in discomfort with CO_2_ with also a smaller increase in waist circumference due to insufflation.^51^ Nitrous oxide gas has been proposed as a comparable alternative or addition to intravenous analgesia in patients undergoing colonoscopy. Welchman et al. performed a systematic review of eleven studies and 623 patients, which reported nitrous oxide is comparable in analgesia to intravenous sedation. The rapid recovery post procedure also enables quicker patient discharge and removes the need of a chaperone on discharge, thus increasing the access to colonoscopy.^52^

Magnetic endoscopic imaging (MEI) such as ‘Scope guide’ (Olympus Medical) or ‘Scope pilot’ (Pentax Medical) has been a major factor in trainee endoscopists becoming competent in colonoscopy at a quicker rate. A prospective study with 102 patients and a meta-analysis of eight randomised control trials and 2967 patients reported improved pain control and intubation rate. Image guidance facilitating the straightening of loops within the sigmoid and improved pain scores for non-experienced endoscopists.^28,53^

Water immersion and water exchange colonoscopy are building a strong evidence base. They have been associated with reduced intraprocedural discomfort with no significant differences in quality of bowel preparation, caecal intubation time, withdrawal time, adenoma detection rate, and postprocedural complications.^54^ Water immersion (WI) is when water is infused during insertion and water removed during withdrawal. Water exchange (WE) is when you use water infusion with water removal primarily during insertion. Compared with carbon dioxide, water insufflation has shown to be significantly lower in mean pain score as demonstrated in an experimental study focusing on patients anticipated to have a difficult colonoscopy.^55^ A qualitative systematic review comparing air to both WI and WE reported a significant reduction in pain and discomfort. It also reported WE may be superior to WI in reducing pain and discomfort alongside also increasing adenoma detection rate (ADR).^56–58^

Abdominal pressure when applied effectively can help shorten the length of the procedure, minimise the angle of turns in the colon and minimise discomfort to the patient.

Crocket et al. studied the effect of a lower abdominal compression device (ColoWrap) in a randomised control trial of 350 patients.^59^ It showed not only improved satisfaction from the patient’s perspective but also healthcare workers; with less discomfort reported from staff having to apply less manual abdominal pressure. Newer forms are in production in the form of abdominal binders which go on the patient similar to a belt strap.^32^

Cap-assisted colonoscopy has been reported in studies to not only improve caecal intubation rates and polyp detection but also reduce patient discomfort. The technique uses a small transparent cap attached to the distal end of the colonoscope. Studies on unsedated patients reported a significantly lower insertion pain score. A study also reported further improvement when used in combination with WE.^60^

### Other factors

Patients who undergo bidirectional endoscopies are more likely to experience discomfort during their colonoscopy following a gastroscopy. It is hypothesised that increased luminal distention due to air or CO_2_ insufflation at gastroscopy as well as stretch of mesenteric attachments are causative factors.^61^

Adequate bowel preparation is strongly associated with reduced pain and difficulty in completing a procedure. It also results in a shorter procedure duration.^15^ A study reported patients are often better prepared when scheduled for morning procedures, in addition, afternoon procedures have been correlated with poorer colon preparation. The study also suggested after accounting for preparation, incomplete colonoscopies were greater in the afternoon possibly due to operator fatigue.^24^

The total duration of a procedure may be influenced by the skill of the endoscopist with a shorter duration associated with reduced discomfort and a longer duration a strong predictor of pain during colonoscopy. Failure to reach the caecum with a low caecal intubation rate was also indicative of a higher level of discomfort. Difficulty maintaining air insufflation alongside over insufflation with overstretching of the mucosa can also increase discomfort.^24^

## New and novel techniques to reduce pain associated with colonoscopy

**Figure 3.**
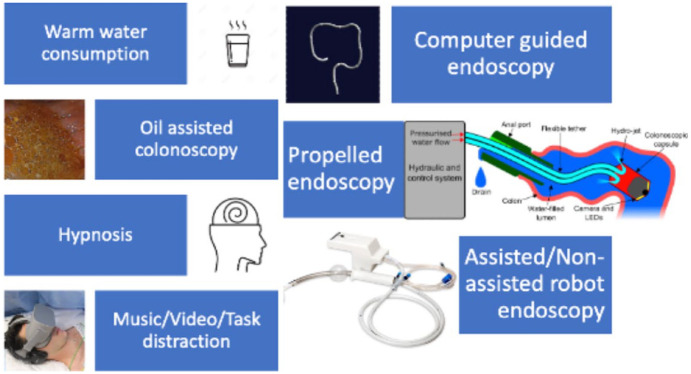
Summary of new and novel techniques being used to improve discomfort.

Several novel concepts have been introduced to aid and improve discomfort (Figure 3). There is, however, little data, and further work is required to allow clinical translation and implementation into everyday practice with improvement in ADR and intubation rates still required.

Robot endoscopy systems offer a novel approach to device insertion and withdrawal, which could aid in reducing discomfort. EndoDrive was the first commercially available system for electro-mechanical support of insertion. The system allows positioning and driving of the endoscope shaft with a foot pedal leaving both hands free for more accurate manoeuvring. Rotation is however still done manually.^62^ Endotics is the first system applied in clinical practice; it consists of a disposable electro-pneumatic self-advancing remote-controlled colonoscope, which uses a clamper mechanism similar to a worm.

Patient discomfort could be further improved with removal of any manually applied pressure with non-assisted robot endoscopy. Examples include The Aer-O-Scope system; a pneumatically actuated disposable colonoscope using two balloons to achieve forward movement. The Invendoscope is a motor-controlled colonoscope where movement is achieved by an inverted sleeve driving unit with wheels that grip onto the inverted sleeve and propel the endoscope forwards or backwards.^62^

NeoGuide is an example of computer-guided endoscopy, which utilises a ‘fully articulated, computer-controlled insertion tube’.^63^ The segments of the device follow each other, and sensors allow tip position and insertion depth to be monitored. The device has reported a reduction in looping, and pain during colonoscopy on the eleven patients tested.

Hydro-colonoscopy is another assisted example that uses a propelled endoscopy device that navigates through a water-filled colon with propelled jets and a tethered capsule.^64^ A pilot study showed successful navigation through setups including alpha loops and reverse alpha loops with difficulty negotiating the N loop. Specific discomfort was not reported on, but initial results suggest further development towards clinical translation is worthwhile.

Further novel approaches include consumption of warm water before colonoscopy, oil-assisted technique and hypnosis have been studied. All these techniques demonstrated a significant reduction in pain.^65–67^ In addition, both the water consumption technique and oil-assisted technique also improved caecal intubation rate. Hypnosis has been reported to reduce the need for sedation and recovery time. In one study no significant difference in discomfort was seen in the hypnosis group compared to standard colonoscopy but did report a greater improvement in anxiety in patients.^68^

Changing the endoscopy room environment and patient role with audio, video and task distraction is a growing area of intervention. The most established of these is music, which is played in the endoscopy room during the procedure. A meta-analysis of RCTs with just under 1000 patients showed a statistically significant reduction in procedure time and pain reported. No statistically significant differences were observed between music and no music during colonoscopy for midazolam.^69^

A newer format is distraction with tasks, video and virtual reality (VR). Video multimedia health informatics have been shown to reduce examination-related anxiety and pain. The use of VR headsets was incorporated in an RCT where patients in the experimental arm undertook VR application tasks and this showed a significant improvement in pain scores.^70–73^

## Discussion

Patient discomfort in some degree is unavoidable; the physiological mechanisms involved in performing a colonoscopy with bowel distention and ligament stretching is required to achieve caecal intubation. Geographical differences do exist in endoscopy when it comes to technique, patient cohorts and sedation selection, and needs to be taken into consideration when discussing pain.

This review is also mainly applicable to health settings that apply unsedated or minimally sedated colonoscopy and without access to propofol-assisted colonoscopy. General anaesthetic-assisted colonoscopy will be more likely in private settings and in many developed countries outside the UK such as the USA.

The implementation of these new techniques outside the UK has also been described. In Europe with robotic-assisted colonoscopy such as Endotics, which was developed in Italy and achieved Food and Drug Administration (FDA) and European Conformity (CE) approval. Water assistance and exchange has also been progressed in these regions alongside the USA with prospective studies. It has also been a staple of routine technique in eastern clinical settings. Novel methods of hypnosis are being led by studies from the USA and oil assistance and lubrication in colonoscopy studies by European research centres.

We have discussed non-modifiable patient factors such as previous abdominal surgery such as a hysterectomy which have been shown to increase the likelihood of patient discomfort and are unavoidable. One study described a history of colorectal resection was inversely related to discomfort and raises the debate of increased risk of adhesions causing discomfort versus a shorter colon reducing the events of discomfort.^24^

Endoscopic technique has played a pivotal role in comfort; better awareness of ergonomics and patient position helps improve the quality of colonoscopy and reducing patient discomfort. Torque steering should be highlighted as a key part of learning to perform colonoscopy and again has allowed the bowel wall to be traversed in a smooth manner. A head-to-head study comparing patient discomfort using Pentax to Olympus colonoscopes reported a lower rate of discomfort in the latter.^74^

There are also devices and technologies to improve the time taken to achieve a caecal intubation, improve the quality of bowel assessment and decrease patient pain. Some of the most effective and simple measures have included variable stiffness and distal attachments to the colonoscope.

Modifying the factors with aids has shown to decrease intubation times and duration of looping and increase completion rates.^28,53^ Interestingly, there have been opposing studies that have shown no improvement in caecal intubation rate with the use of this technology. Some of the novel techniques such as WI endoscopy allow cheap and accessible options to help improve discomfort.

Robotic assistance and aids are also in development. Many are inspired from animal locomotion aiming to reduce its comfort in navigating the large bowel. This is exciting work and is gaining popularity. A novel non-assisted automated device that performs safe and effective colonoscopy whilst minimising patient discomfort is a research priority.

The cost-effectiveness of new technologies such as robotic endoscopic systems and VR applications requires further study. Initial high upfront costs to implement new technology may be a deterrent to clinical units and may first be seen in academic centres who can support the initial financial investment. A detailed report outlining the long-term savings will need to be demonstrated to translate these novel solutions into routine daily clinical care for patients.

Regional bias in data and further evaluation of the practicality of new technologies will need to be a focus of future research to find solutions to these challenges and ensure true implementation into practice.

This paper has outlined the factors contributing alongside aids, solutions and modifications to improve discomfort. These are some recommendations to minimise discomfort and pain associated with colonoscopy based on studies discussed in this review:

1. Sedation should be titrated according to patient factors including comorbidity.
2. Sedation is recommended for young females and patients with anxiety, history of hysterectomy and diverticular disease.
3. Consider the use of a thinner than standard scope for low BMI and shorter patients.
4. Consider utilising variable stiffness or MEI if available to help prevent loop formation.
5. The technique of WE should be considered to decrease discomfort.

## Conclusion

Pain is a major burden for patients undergoing colonoscopy and can affect patient experience and outcomes. It also impacts the uptake of colonoscopy in general and for follow-up surveillance procedures.

There are modifiable and non-modifiable factors associated with pain during colonoscopy. These factors can guide novel research to identify solutions that can improve the patient experience and decrease discomfort.

The task of improving discomfort in colonoscopy is multi-faceted. The design of the endoscope, the operator skill and aids used in colonoscopy are paramount. They play a vital role in achieving a high-quality comfortable colonoscopy for the patient.

## Declarations

**Ethics approval and consent to participate:** Not applicable.

**Consent for publication:** Not applicable.

**Availability of data and materials:** Not applicable.
